# Dissemination and Implementation of a Community Health Worker Intervention for Disparities in Palliative Care (DeCIDE PC): a study protocol for a hybrid type 1 randomized controlled trial

**DOI:** 10.1186/s12904-023-01250-0

**Published:** 2023-09-18

**Authors:** Amn Siddiqi, Olivia Monton, Alison Woods, Taleaa Masroor, Shannon Fuller, Jill Owczarzak, Gayane Yenokyan, Lisa A. Cooper, Karen M. Freund, Thomas J. Smith, Jean S. Kutner, Kathryn L. Colborn, Robert Joyner, Ronit Elk, Fabian M. Johnston

**Affiliations:** 1 Department of Surgery, Johns Hopkins University School of Medicine, 600 N Wolfe Street, Baltimore, MD 21287 USA; 2 Department of Epidemiology, Johns Hopkins Bloomberg School of Public Health, 615 N Wolfe Street, Baltimore, MD 21205 USA; 3 Department of Health, Behavior and Society, Johns Hopkins Bloomberg School of Public Health, 624 N Broadway, Baltimore, MD 21205 USA; 4 Department of Biostatistics, Johns Hopkins Bloomberg School of Public Health, 615 N Wolfe Street, Baltimore, MD 21205 USA; 5 Johns Hopkins University School of Nursing, 525 N Wolfe Street, Baltimore, MD 21205 USA; 6 Department of Medicine, Johns Hopkins University School of Medicine, 2024 East Monument Street, Suite 2-515, Baltimore, MD 21287 USA; 7 Department of Medicine, Tufts University School of Medicine, 800 Washington Street, Boston, MA 02111 USA; 8 Department of Medicine, Johns Hopkins University School of Medicine, 600 N Wolfe Street, Baltimore, MD 21287 USA; 9 Department of Medicine, University of Colorado School of Medicine, 12401 E 17th Ave, Aurora, CO 80045 USA; 10 Department of Medicine, School of Medicine, University of Colorado Anschutz Medical Campus, 13001 E 17th Place, Aurora, CO 80045 USA; 11 Richard A. Henson Research Institute, TidalHealth Peninsula Regional, 100 East Carroll Street, Salisbury, MD 21801 USA; 12 Department of Medicine, University of Alabama at Birmingham, 933 19th Street S, Birmingham, AL 35205 USA; 13 Division of Surgical Oncology, Department of Surgery, Johns Hopkins University, 600 N Wolfe Street, Blalock 606, Baltimore, MD 21287 USA

**Keywords:** Palliative care, Cancer, Advanced cancer, Community health worker, CHW, Community, Health disparities, Racial disparities

## Abstract

**Background:**

There are persistent racial and ethnic health disparities in end-of-life health outcomes in the United States. African American patients are less likely than White patients to access palliative care, enroll in hospice care, have documented goals of care discussions with their healthcare providers, receive adequate symptom control, or die at home. We developed Community Health Worker Intervention for Disparities in Palliative Care (DeCIDE PC) to address these disparities. DeCIDE PC is an integrated community health worker (CHW) palliative care intervention that uses community health workers (CHWs) as care team members to enhance the receipt of palliative care for African Americans with advanced cancer. The overall objectives of this study are to (1) assess the effectiveness of the DeCIDE PC intervention in improving palliative care outcomes amongst African American patients with advanced solid organ malignancy and their informal caregivers, and (2) develop generalizable knowledge on how contextual factors influence implementation to facilitate dissemination, uptake, and sustainability of the intervention.

**Methods:**

We will conduct a multicenter, randomized, assessor-blind, parallel-group, pragmatic, hybrid type 1 effectiveness-implementation trial at three cancer centers across the United States. The DeCIDE PC intervention will be delivered over 6 months with CHW support tailored to the individual needs of the patient and caregiver. The primary outcome will be advance care planning. The treatment effect will be modeled using logistic regression. The secondary outcomes are quality of life, quality of communication, hospice care utilization, and patient symptoms.

**Discussion:**

We expect the DeCIDE PC intervention to improve integration of palliative care, reduce multilevel barriers to care, enhance clinic and patient linkage to resources, and ultimately improve palliative care outcomes for African American patients with advanced cancer. If found to be effective, the DeCIDE PC intervention may be a transformative model with the potential to guide large-scale adoption of promising strategies to improve palliative care use and decrease disparities in end-of-life care for African American patients with advanced cancer in the United States.

**Trial registration:**

Registered on ClinicalTrials.gov (NCT05407844). First posted on June 7, 2022.

## Introduction

### Background and rationale

Palliative care is an interdisciplinary approach that aims to improve the quality of life (QOL) of patients with advanced stage illnesses and their families [1]. The American Society of Clinical Oncology (ASCO) recommends early integration of palliative care for all patients with advanced cancer [2]. This approach enables early identification and management of distressing physical, psychosocial, and spiritual issues, facilitates communication and support throughout decision-making processes, and enhances the receipt of goal-concordant care [3, 4].

There are persistent racial and ethnic health disparities in end-of-life health outcomes in the United States. African American patients are less likely than White patients to access or receive palliative care, receive adequate pain control, or enroll in hospice services, all of which results in unnecessary suffering at the end-of-life [5–7]. Physician or patient-initiated referral to palliative care is the standard of cancer care for patients with advanced cancer in the United States, but stark and worsening disparities in outcomes suggest this model may be inadequate for African Americans, especially when considering cultural influences and underlying social determinants of health (SDOH). The totality of these disparities results in African American patients failing to receive goal-concordant end-of-life care [5, 6, 8, 9].

Multiple randomized controlled trials have demonstrated the benefit of diverse interventions to improve palliative care utilization; however, few have been implemented [10, 11]. Literature often fails to comprehensively account for multilevel barriers when evaluating existing palliative care programs. Moreover, the lack of racial and ethnic representation in existing studies fails to provide insight into the role of culture and underlying SDOH in palliative care utilization [8, 12]. Furthermore, the opinions and needs of stakeholders (patients, caregivers, oncologists, palliative care providers, and cancer center leaders) have not been adequately addressed and require further evaluation. Lastly, successful studies often fail to consider the role of implementation or dissemination of their findings, which perpetuates the lack of quality palliative and end-of-life care [13].

Community health workers (CHWs) are non-clinician public health workers who can improve care consistency by addressing SDOH and helping patients from underserved communities overcome barriers to health care. Numerous studies have demonstrated the ability of CHWs to decrease care disparities across multiple levels, settings, and diseases [14–16]. CHWs bridge the gap between communities and the healthcare system by delivering culturally sensitive and contextually appropriate care. This has been shown to improve healthcare engagement, self-management, self-efficacy, treatment plan adherence, and health outcomes [17–19].

We developed an integrated community health worker (CHW) palliative care intervention, Community Health Worker Intervention for Disparities in Palliative Care (DeCIDE PC). The DeCIDE PC intervention is a theory-driven, stakeholder-informed palliative care intervention, which utilizes CHWs as care team members to enhance the receipt of palliative care for African Americans with advanced cancer [20, 21]. In this role, CHWs may help patients and their families overcome barriers in the adoption of palliative care services by improving provision of non-physician support services, enhancing palliative care education, helping patients navigate the healthcare system, advocating for patients and their families within the healthcare setting, and empowering patients to discuss goals of care and advance care planning (ACP) with their families and care teams. A pilot study demonstrated that this intervention was acceptable, feasible, and effective at mitigating the adverse impact of SDOH and improving palliative care outcomes, such as completion of advance directives. The collective experience of our study team highlights the potential benefit of the DeCIDE PC intervention and supports further investigation [20, 21].

### Objectives

The overall objectives of this study are to:

1. Assess the effectiveness of the DeCIDE PC intervention in improving palliative care outcomes amongst African American patients with advanced solid organ malignancy and their informal caregivers, and
2. Develop generalizable knowledge on how contextual factors influence implementation to facilitate dissemination, implementation, and sustainability of the intervention.

These objectives will be accomplished through three specific aims, which are to:

1. Refine the DeCIDE PC intervention to address multilevel implementation barriers,
2. Compare the effectiveness of the DeCIDE PC intervention to enhanced standard of care in improving palliative care outcomes, and
3. Evaluate the implementation of the DeCIDE PC intervention.

We hypothesize that the DeCIDE PC intervention will improve ACP (primary outcome), QOL (principal secondary outcome), and other palliative care outcomes in African American patients with advanced cancer. Further, we anticipate that the findings from this project will inform wider implementation and scale-up of the DeCIDE PC intervention.

## Methods

This study protocol was written in accordance with the Standard Protocol Items: Recommendations for Interventional Trials (SPIRIT) statement [22] (Tables 1 and 2).

**Table 1:** SPIRIT 2013 Checklist

Section/Item	Item no	Description	**Item reported**
**Administrative information**			
Title	1	Descriptive title identifying the study design, population, interventions, and, if applicable, trial acronym	Yes
Trial registration	2a	Trial identifier and registry name. If not yet registered, name of intended registry	Yes
	2b	All items from the World Health Organization Trial Registration Data Set	Yes (Table 2)
Protocol version	3	Date and version identifier	Yes
Funding	4	Sources and types of financial, material, and other support	Yes
Roles and responsibilities	5a	Names, affiliations, and roles of protocol contributors	Yes
	5b	Name and contact information for the trial sponsor	Yes
	5c	Role of study sponsor and funders, if any, in study design; collection, management, analysis, and interpretation of data; writing of the report; and the decision to submit the report for publication, including whether they will have ultimate authority over any of these activities	Yes
	5d	Composition, roles, and responsibilities of the coordinating centre, steering committee, endpoint adjudication committee, data management team, and other individuals or groups overseeing the trial, if applicable (see Item 21a for data	Yes
**Introduction**			
Background and rationale	6a	Description of research question and justification for undertaking the trial, including summary of relevant studies (published and unpublished) examining benefits and harms for each intervention	Yes
	6b	Explanation for choice of comparators	Yes
Objectives	7	Specific objectives or hypotheses	Yes
Trial design	8	Description of trial design including type of trial (eg, parallel group, crossover, factorial, single group), allocation ratio, and framework (eg, superiority, equivalence, noninferiority, exploratory)	Yes
**Methods: Participants, interventions, and outcomes**			
Study setting	9	Description of study settings (eg, community clinic, academic hospital) and list of countries where data will be collected. Reference to where list of study sites can be obtained	Yes
Eligibility criteria	10	Inclusion and exclusion criteria for participants. If applicable, eligibility criteria for study centres and individuals who will perform the interventions (eg, surgeons, psychotherapists)	Yes
Interventions	11a	Interventions for each group with sufficient detail to allow replication, including how and when they will be administered	Yes
	11b	Criteria for discontinuing or modifying allocated interventions for a given trial participant (eg, drug dose change in response to harms, participant request, or improving/worsening disease)	Yes
	11c	Strategies to improve adherence to intervention protocols, and any procedures for monitoring adherence (eg, drug tablet return, laboratory tests)	Yes
	11d	Relevant concomitant care and interventions that are permitted or prohibited during the trial	Yes
Outcomes	12	Primary, secondary, and other outcomes, including the specific measurement variable (eg, systolic blood pressure), analysis metric (eg, change from baseline, final value, time to event), method of aggregation (eg, median, proportion), and time point for each outcome. Explanation of the clinical relevance of chosen efficacy and harm outcomes is strongly recommended	Yes
Participant timeline	13	Time schedule of enrolment, interventions (including any run-ins and washouts), assessments, and visits for participants. A schematic diagram is highly recommende	Yes
Sample size	14	Estimated number of participants needed to achieve study objectives and how it was determined, including clinical and statistical assumptions supporting any sample size calculations	Yes
Recruitment	15	Strategies for achieving adequate participant enrolment to reach target sample size	Yes
**Methods: Assignment of interventions (for controlled trials)**			
Allocation:			
Sequence generation	16a	Method of generating the allocation sequence (eg, computer-generated random numbers), and list of any factors for stratification. To reduce predictability of a random sequence, details of any planned restriction (eg, blocking) should be provided in a separate document that is unavailable to those who enrol participants or assign interventions	Yes
Allocation concealment mechanism	16b	Mechanism of implementing the allocation sequence (eg, central telephone; sequentially numbered, opaque, sealed envelopes), describing any steps to conceal the sequence until interventions are assigned	Yes
Implementation	16c	Who will generate the allocation sequence, who will enrol participants, and who will assign participants to interventions	Yes
Blinding (masking)	17a	Who will be blinded after assignment to interventions (eg, trial participants, care providers, outcome assessors, data analysts), and how	Yes
	17b	If blinded, circumstances under which unblinding is permissible, and procedure for revealing a participant’s allocated intervention during the trial	Yes
**Methods: Data collection, management, and analysis**			
Data collection methods	18a	Plans for assessment and collection of outcome, baseline, and other trial data, including any related processes to promote data quality (eg, duplicate measurements, training of assessors) and a description of study instruments (eg, questionnaires, laboratory tests) along with their reliability and validity, if known. Reference to where data collection forms can be found, if not in the protocol	Yes
	18b	Plans to promote participant retention and complete follow-up, including list of any outcome data to be collected for participants who discontinue or deviate from intervention protocols	Yes
Data management	19	Plans for data entry, coding, security, and storage, including any related processes to promote data quality (eg, double data entry; range checks for data values). Reference to where details of data management procedures can be found, if not in the protocol	Yes
Statistical methods	20a	Statistical methods for analysing primary and secondary outcomes. Reference to where other details of the statistical analysis plan can be found, if not in the protocol	Yes
	20b	Methods for any additional analyses (eg, subgroup and adjusted analyses)	Yes
	20c	Definition of analysis population relating to protocol non-adherence (eg, as randomised analysis), and any statistical methods to handle missing data (eg, multiple imputation)	Yes
**Methods: Monitoring**			
Data monitoring	21a	Composition of data monitoring committee (DMC); summary of its role and reporting structure; statement of whether it is independent from the sponsor and competing interests; and reference to where further details about its charter can be found, if not in the protocol. Alternatively, an explanation of why a DMC is not needed	Yes
	21b	Description of any interim analyses and stopping guidelines, including who will have access to these interim results and make the final decision to terminate the trial	Yes
Harms	22	Plans for collecting, assessing, reporting, and managing solicited and spontaneously reported adverse events and other unintended effects of trial interventions or trial conduct	Yes
Auditing	23	Frequency and procedures for auditing trial conduct, if any, and whether the process will be independent from investigators and the sponsor	Yes
**Ethics and dissemination**			
Research ethics approval	24	Plans for seeking research ethics committee/institutional review board (REC/IRB) approval	Yes
Protocol amendments	25	Plans for communicating important protocol modifications (eg, changes to eligibility criteria, outcomes, analyses) to relevant parties (eg, investigators, REC/IRBs, trial participants, trial registries, journals, regulators)	Yes
Consent or assent	26a	Who will obtain informed consent or assent from potential trial participants or authorised surrogates, and how (see Item 32)	Yes
	26b	Additional consent provisions for collection and use of participant data and biological specimens in ancillary studies, if applicable	Yes
Confidentiality	27	How personal information about potential and enrolled participants will be collected, shared, and maintained in order to protect confidentiality before, during, and after the trial	Yes
Declaration of interests	28	Financial and other competing interests for principal investigators for the overall trial and each study site	Yes
Access to data	29	Statement of who will have access to the final trial dataset, and disclosure of contractual agreements that limit such access for investigators	Yes
Ancillary and post-trial care	30	Provisions, if any, for ancillary and post-trial care, and for compensation to those who suffer harm from trial participation	Yes
Dissemination policy	31a	Plans for investigators and sponsor to communicate trial results to participants, healthcare professionals, the public, and other relevant groups (eg, via publication, reporting in results databases, or other data sharing arrangements), including any publication restrictions	Yes
	31b	Authorship eligibility guidelines and any intended use of professional writers	Yes
	31c	Plans, if any, for granting public access to the full protocol, participant-level dataset, and statistical code	Yes
**Appendices**			
Informed consent materials	32	Model consent form and other related documentation given to participants and authorised surrogates	Not applicable
Biological specimens	33	Plans for collection, laboratory evaluation, and storage of biological specimens for genetic or molecular analysis in the current trial and for future use in ancillary studies, if applicable	Not applicable

**Table 2:** World Health Organization Trial Registration Data Set (Version 1.3.1)

Data Item	Information
Primary Registry and Trial Identifying Number	Registered on ClinicalTrials.gov, NCT05407844
Date of Registration in Primary Registry	First posted on June 7, 2022
Secondary Identifying Numbers	Not applicable
Source(s) of Monetary or Material Support	This study is funded by the National Cancer Institute (NCI) Grant number: 1R01CA252101-01A1
Primary Sponsor	The study sponsor is Johns Hopkins University
Secondary Sponsor(s)	Not applicable
Contact for Public Queries	OM (omonton1@jh.edu) TM (tmasroo1@jh.edu)
Contact for Scientific Queries	FJ (fjohnst4@jhmi.edu)
Public Title	Community Health Worker Intervention for Disparities in Palliative Care (DeCIDE PC)
Scientific Title	Dissemination and Implementation of a Community Health Worker Intervention for Disparities in Palliative Care (DeCIDE PC): a study protocol for a hybrid type 1 randomized controlled trial
Countries of Recruitment	United States
Health Condition(s) or Problem(s) Studied	Disparities in the access to and utilization of palliative care among African American patients with advanced solid organ malignancy
Intervention(s)	Intervention group: Community Health Worker Intervention for Disparities in Palliative Care (DeCIDE PC); theory-driven, stakeholder-informed palliative care intervention, which utilizes CHWs as care team members to enhance the receipt of palliative care for African Americans with advanced cancer Comparator group: Enhanced standard of care; standard of care and a palliative care brochure
Key Inclusion and Exclusion Criteria	Inclusion criteria for patients: Adult (≥ 18 years old) patients who (1) self-identify as African American, (2) have advanced solid organ malignancy (AJCC stage III or IV), (3) are English speaking, (4) have intact cognition and an ability to provide informed consent, and (5) have not had any palliative care experience within the last year Exclusion criteria for patients include: (1) age < 18 years old, (2) unable to read or comprehend English, (3) unable to provide informed consent, and (4) palliative care experience within the last year Inclusion criteria for caregivers: Adult (≥ 18 years old) caregivers who (1) provide informal (unpaid) care to an eligible African American cancer patient (related or unrelated), (2) are English speaking, and (3) have intact cognition and an ability to provide informed consent Exclusion criteria for caregivers include: (1) age < 18 years old, (2) unable to read or comprehend English, and (3) unable to provide informed consent
Study Type	Type of study: Interventional Study design: Multicenter, randomized, assessor-blind, parallel-groups, pragmatic, hybrid type 1 effectiveness-implementation trial involving patients from three oncology practices in the United States
Date of First Enrollment	September 2023 (anticipated)
Sample Size	Target sample size: 160 patient-caregiver dyads (total 320 participants)
Recruitment Status	Pending: Participants are not yet being recruited or enrolled at any site
Primary Outcome(s)	Outcome Name: Advance care planning Metric/method of measurement: Self-reported or documented Advance Directive or a documented discussion of care preferences between the patient and caregiver or healthcare team Timepoints: Baseline, 2 months, 6 months
Key Secondary Outcomes	Outcome Name: Quality of life Metric/method of measurement: Quality of Life measured by the Functional Assessment of Chronic Illness Therapy - Palliative Care Timepoints: Baseline, 2 months, 6 months
Ethics Review	Status: Approved Date of approval: March 31, 2022
Completion Date	Not applicable
Summary Results	Not applicable
IPD Sharing Statement	Plan to share IPD: Yes Plan description: We plan to make the full protocol, deidentified participant-level data, and the statistical code available from the corresponding author on reasonable request

### Study design

**Fig. 1:**
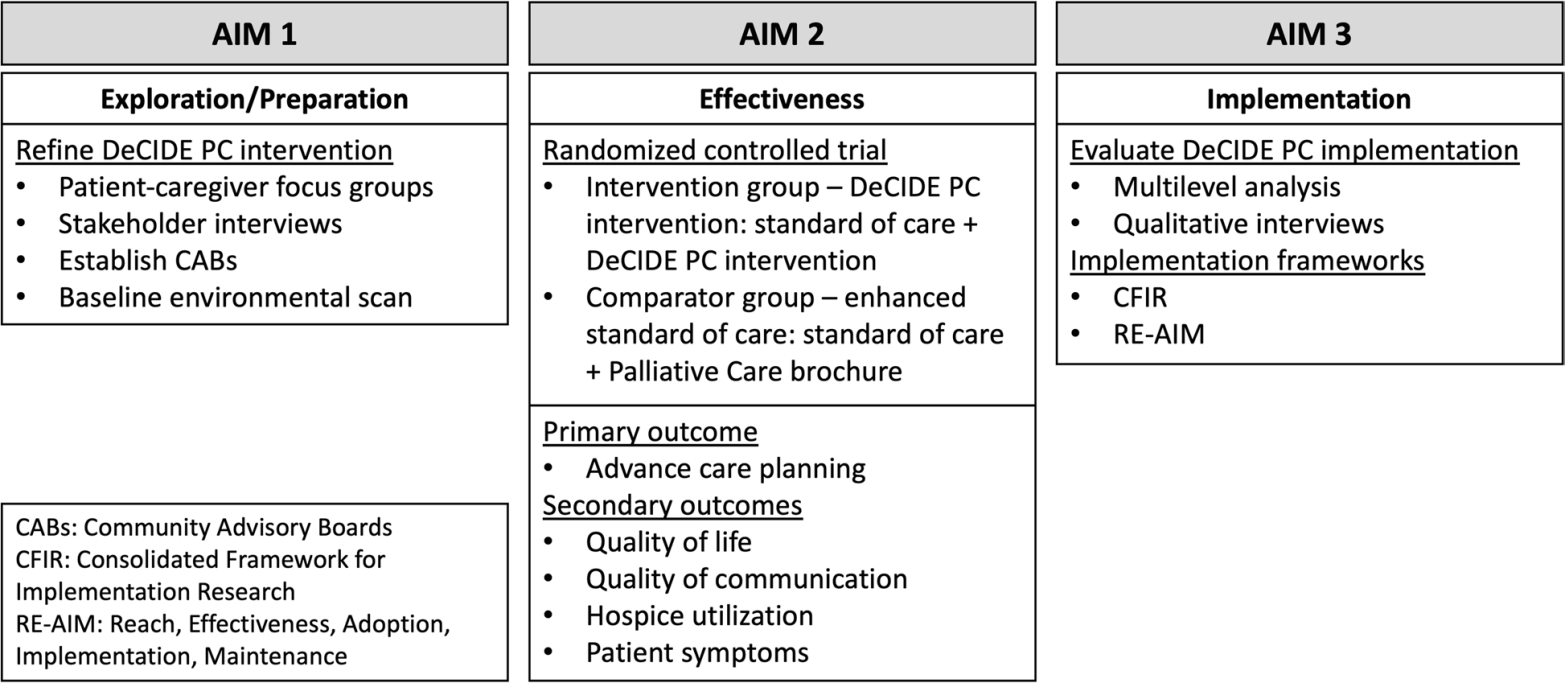
Study design

We will first use a mixed methods approach to refine the DeCIDE PC intervention by addressing multilevel implementation barriers (Aim 1). This stage will involve gathering input from patients and caregivers through patient-caregiver dyad focus groups and eliciting input from stakeholders in oncology, palliative care, and the community through baseline key informant interviews. Additionally, we will initiate annual environmental scans and establish a Community Advisory Board (CAB) at each enrollment site. The site-specific CABs will be comprised of patients, family members, community members, and health system members. We will aim to recruit 8–10 members at each site. The CABs will meet quarterly and will be asked to review and provide feedback on recruitment and retention approaches, data collection procedures, intervention content and delivery, interpretation of results, and dissemination of findings. Following Aim 1, we will evaluate the effectiveness of the DeCIDE PC intervention in improving palliative care outcomes in African American patients with advanced cancer and their informal caregivers compared to enhanced standard of care (Aim 2). This will be accomplished through a multicenter, randomized, assessor-blind, parallel-group, pragmatic, hybrid type 1 effectiveness-implementation trial with a 1:1 allocation ratio. Finally, we will use a mixed methods approach and employ the CFIR (Consolidated Framework for Implementation Research) and RE-AIM (Reach, Effectiveness, Adoption, Implementation, Maintenance) frameworks to evaluate implementation of the DeCIDE PC intervention (Aim 3). This will be achieved through multilevel analysis of intervention delivery and qualitative interviews. An overview of the study design is outlined in Fig. 1. This paper will focus on the protocol related to Aim 2, the randomized controlled trial.

### Study setting

This study will take place at three cancer centers across the United States: Johns Hopkins Hospital (Broadway and Bayview campuses, Baltimore, Maryland), the University of Alabama at Birmingham Hospital (Birmingham, Alabama), and TidalHealth Peninsula Regional (Salisbury, Maryland). These sites were strategically chosen as they represent different socioeconomic, cultural, and demographic features of the African American community, and have established CHW programs.

### Eligibility criteria

**Fig. 2:**
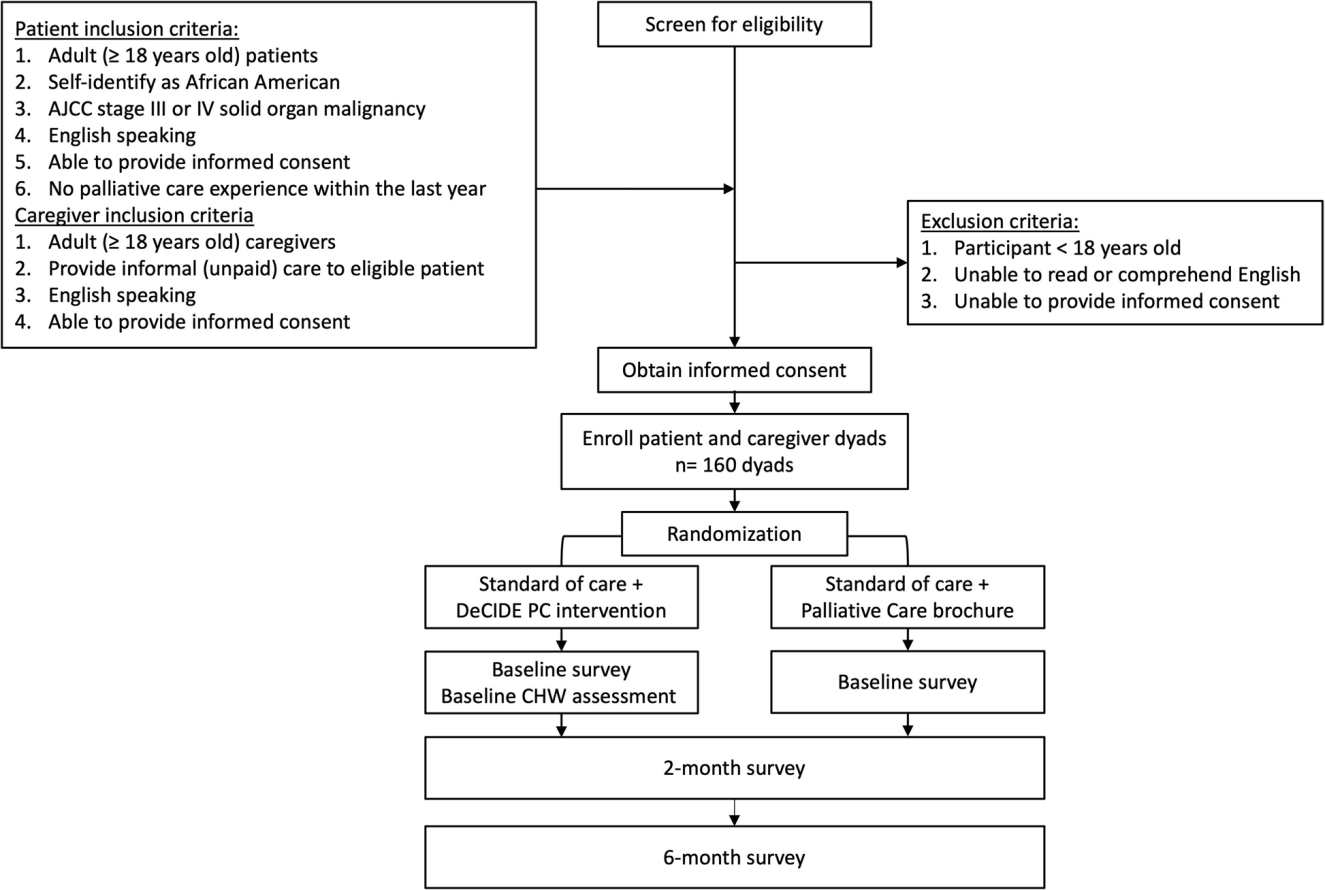
Trial flow diagram

The intervention will target patients with advanced cancer and their self-designated informal caregivers (patient-caregiver dyads). Eligibility criteria for patients: Adult (≥ 18 years old) patients who (1) self-identify as African American, (2) have advanced solid organ malignancy (AJCC stage III or IV), (3) are English speaking, (4) have intact cognition and an ability to provide informed consent, and (5) have not had any palliative care experience within the last year. Exclusion criteria for patients include: (1) age < 18 years old, (2) unable to read or comprehend English, (3) unable to provide informed consent, and (4) palliative care experience within the last year. Eligibility criteria for caregivers: Adult (≥ 18 years old) caregivers who (1) provide informal (unpaid) care to an eligible African American cancer patient (related or unrelated), (2) are English speaking, and (3) have intact cognition and an ability to provide informed consent. Exclusion criteria for caregivers include: (1) age < 18 years old, (2) unable to read or comprehend English, and (3) unable to provide informed consent. In instances where no caregiver is available or willing to participate, patients will remain eligible to participate in the study individually. The participant flowchart is outlined in Fig. 2.

### Informed consent

Study coordinators will approach potential participants in-person or via telephone to discuss the study goals, risks, and alternatives, and obtain oral and written informed consent. Participants will identify a caregiver and a separate informed consent will be obtained.

### Additional consent provisions for collection and use of participant data and biological specimens

There are no additional consent provisions for this study.

### Intervention description

#### Intervention group – DeCIDE PC intervention

Participants in the intervention group will receive the DeCIDE PC intervention in addition to standard of care. Following study enrollment, participants randomized to the DeCIDE PC intervention group will be connected with the CHW at their enrollment site. The CHWs will then meet with the patient-caregiver dyad or the patient alone to perform a baseline assessment of their SDOH and barriers to healthcare. Following the baseline assessment, CHWs will perform weekly check-ins and tailor ongoing support to the individual needs of the patient and caregiver. CHWs will have access to the electronic medical record (EMR) at their respective sites to follow their patients’ course of care and communicate with the clinical team through patient notes and direct messaging. The CHW intervention activities will include educating patients on palliative and hospice care, facilitating palliative and hospice care referrals and care team communications, linking patients to community-based resources, empowering patients to discuss their goals of care with their oncologists and encouraging patients to discuss advance care planning with their caregivers and families, removing barriers to care by addressing SDOH, and providing instrumental, logistical, and emotional support [21, 23, 24]. This intervention will be refined based on the findings from Aim 1, which will include input from patients and caregivers, stakeholders, the CAB, and the baseline environmental scan.

We will hire and train one CHW at each enrollment site in the first year of the study. All CHWs will undergo a three-month CHW training program, which will include a combination of synchronous (didactic and problem-based sessions, communication and motivational interviewing skills training), asynchronous, and experiential training components. For the purposes of program evaluation, all CHWs will complete pre- and post-training tests to assess knowledge gain, perceived competence, and satisfaction with the training. CHWs will also undergo longitudinal training throughout the study period. We will also onboard a Palliative Care Liaison at each enrollment site, who will be a member of the palliative care team and will serve as a resource and source of support for the CHW throughout the study. Finally, CHWs will have access to up-to-date palliative care content from national organizations, such as the Center to Advance Palliative Care (CAPC).

### Choice of comparator

#### Comparator group – enhanced standard of care

Participants in the comparison group will receive standard of care in accordance with national and local guidelines for management of their disease. Due to the many known benefits of palliative care for patients with advanced cancer, a brochure on palliative care and its service offerings will also be provided to patients within the enhanced standard of care pathway at the time of enrollment. Additionally, patients may be referred to specialty palliative care at any time throughout the study period at their oncologist’s discretion.

### Criteria for discontinuing or modifying allocated interventions

We will discontinue the study intervention at the participant’s request.

### Strategies to improve adherence to interventions

To improve adherence to the intervention and survey completion, participants in each dyad will receive monetary compensation ($50 per participant, $100 per dyad) upon completion of the 6-month survey. If the patient passes away prior to administration of the 6-month survey, the patient’s caregiver or family will receive the monetary compensation.

### Relevant concomitant care permitted or prohibited during the trial

Patients will be permitted to receive oncologic care throughout the trial. Oncologists may refer patients in the enhanced standard of care group to speciality palliative care, however, to prevent crossover between groups throughout the trial, cannot refer patients to CHW support. Participants will be prohibited from participating in concomitant clinical trials in palliative care.

### Provisions for post-trial care

We do not anticipate that participants will suffer harm from trial participation, and therefore do not anticipate the need for additional compensation or ancillary care. Patients will continue to receive oncological standard of care following study completion.

### Outcomes

The primary, secondary, and exploratory outcomes are summarized in Table 3. The primary outcome for this study is ACP, defined as a self-reported or documented Advance Directive, such as a living will (LW) or durable power of attorney (DPOA), or a documented discussion of care preferences between the patient and caregiver or the patient and the healthcare team. ACP has been widely used and validated in palliative care studies [25, 26]. Our principal secondary outcome is QOL, measured by the Functional Assessment of Chronic Illness Therapy - Palliative Care (FACIT-Pal) [27]. FACIT-Pal contains five subscales, including physical, social/family, emotional, and functional wellbeing, as well as palliative care. Participants will be provided with a list of statements and asked to rate each statement on a scale of 0 (not at all) to 4 (very much) based on their recollection of the last 7 days. The subscale scores will then be added to generate a total score, with higher scores reflecting a higher QOL. FACIT-Pal has also undergone extensive validation in palliative care studies [27, 28]. Other secondary outcomes include (1) Quality of Communication (QOC), measured by the QOC Questionnaire [29]; (2) hospice care utilization within 14 days of death; and (3) patient symptoms, measured by the Edmonton Symptom Assessment Scale (ESAS) [30] and the Center for Epidemiologic Studies Depression Scale (CES-D) [31].

**Table 3:** Overview of primary, secondary, and exploratory outcome measures

Outcome measure	Description
**Primary outcome**	
Advance care planning	Defined as a self-reported or documented Advance Directive, such as a living will (LW) or durable power of attorney (DPOA), or a documented discussion of care preferences between the patient and caregiver or healthcare team
**Secondary outcomes**	
Quality of life	Functional Assessment of Chronic Illness Therapy - Palliative Care (FACIT-Pal) [27]
Quality of communication	Quality of Communication (QOC) Questionnaire [29]
Hospice care utilization	Utilization of hospice care within 14 days of death (Yes/No)
Patient symptoms	Edmonton Symptom Assessment Scale (ESAS) [30] Center for Epidemiologic Studies Depression Scale (CES-D) [31]
**Exploratory outcomes**	
Patient physician communication	Princess Margaret Hospital Satisfaction with Doctor Questionnaire (PMH/PSQ-MD) [32, 33]
Caregiver satisfaction	Family Satisfaction with Advanced Cancer Care (FAMCARE) [34]
Palliative and hospice care utilization	Palliative care consulted (Yes/No), hospice care referral made (Yes/No), length of stay in hospice care (days), hospice care withdrawal (Yes/No)
Resource utilization	Length of stay in hospital (days), length of stay in the Intensive Care Unit (days), visits to the Emergency Department (number of visits), readmission(s) (Yes/No), timing of readmissions (days since discharge), time spent with CHW (hours), cost (dollars)
Social determinants of health	Protocol for Responding to & Assessing Patients' Assets, Risks, & Experiences (PRAPARE) [35]

### Participant timeline

The timetable for data collection is presented in Table 4. Research coordinators from each enrollment site will screen potential participants for eligibility, obtain informed consent, enroll patients and their caregivers, and perform random allocation. They will then conduct EMR review and administer surveys at baseline (t_0_), 2 months (t_1_), and 6 months (t_2_).

**Table 4:** Timetable for data collection

	**STUDY PERIOD**				
	**Enrollment**	**Allocation**	**Baseline (t**_**0**_**)**	**2 months (t**_**1**_**)**	**6 months (t**_**2**_**)**
**Eligibility screen**	X				
**Informed consent**	X				
**Allocation**		X			
**Demographic information**			X		
**Medical comorbidities**			X		
**Performance status**			X		
**Disease characteristics**			X		
**ACP**^**a**^			X	X	X
**FACIT-Pal**^**b**^			X	X	X
**QOC**^**c**^			X	X	X
**Hospice care utilization**			X	X	X
**ESAS**^**d**^			X	X	X
**CES-D**^**e**^			X	X	X
**PMH/PSQ-MD**^**f**^			X	X	X
**FAMCARE**^**g**^			X	X	X
**Palliative and hospice care utilization**			X	X	X
**Resource utilization**			X	X	X
**PRAPARE**^**h**^			X	X	X

^a^*ACP* Advance care planning

^b^*FACIT-Pal* Functional Assessment of Chronic Illness Therapy - Palliative Care [27]

^c^*QOC* Quality of Communication Questionnaire [29]

^d^*ESAS* Edmonton Symptom Assessment Scale [30]

^e^*CES-D* Center for Epidemiologic Studies Depression Scale [31]

^f^*PMH/PSQ-MD* Princess Margaret Hospital Satisfaction with Doctor Questionnaire [32, 33]

^g^*FAMCARE* Family Satisfaction with Advanced Cancer Care [34]

^h^*PRAPARE* Protocol for Responding to & Assessing Patients' Assets, Risks & Experiences [35]

### Sample size

We calculated our sample size to ensure sufficient power to detect a clinically meaningful difference in both our primary outcome (ACP) and our principal secondary outcome (QOL) between the study arms. With an alpha of 0.05 and power of 90%, we estimate a sample size of 56 participant per study arm to detect a 30 percentage point difference in ACP at 6 months, based on effect sizes from previous studies and conservatively assuming that 30% of patients in the enhanced standard care arm will have ACP [26, 36]. After accounting for a 20% attrition rate over the study period, the adjusted final sample size based on our primary outcome is 70 participants per study arm (140 participants total). The sample size calculation based on our principal secondary outcome of QOL, measured by FACIT-Pal, is based on a recent study, which found that palliative care was associated with a standardized mean difference (SMD) in QOL of 0.46 (95% CI 0.08–0.83) [28]. With an alpha of 0.05 and power of 80%, and after accounting for a 20% attrition rate, we estimate a sample size of 80 participants per study arm (160 participants total) to detect a SMD in QOL of 0.5. To ensure adequate power for both outcomes, we will plan to enroll 160 participants. We will seek to recruit a caregiver alongside each patient as a patient-caregiver dyad, but a lack of caregiver participation will not preclude a patient's enrollment. Our total potential sample size for the trial is therefore 320 participants, including 160 patients and 160 caregivers.

### Recruitment

The three enrollment sites have well established cancer centers, albeit different patient volumes. Based on the patient volumes at the three enrollment sites, we anticipate that 40% of the patient-caregiver dyads (65 dyads) will be recruited from Johns Hopkins Hospital, 35% (55 dyads) from University of Alabama at Birmingham Hospital, and the remaining 25% (40 dyads) from TidalHealth Peninsula Regional. Study recruitment will occur over 2–4 years. The CAB from each enrollment site will provide insight and advice to guide ongoing recruitment.

### Allocation

Patient-caregiver dyads will be randomized in a 1:1 ratio into one of two groups, the DeCIDE PC intervention group or the enhanced standard of care group. To yield balanced groups, the random allocation sequence will be computer-generated with a block size of 6. Randomization will be stratified by cancer stage (AJCC stage III or IV). Allocation will be entered into the Research Electronic Data Capture (REDCap) database by a unblinded statistician at the central study site who is not directly involved in the study design, study conduct, or participant enrollment. Randomization will be performed immediately after enrollment by unblinded research coordinators through REDCap.

### Blinding

The principal investigators and lead statistician will be blinded to participant randomization. Due to the nature of the intervention, trial participants and oncologists will not be blinded. Additionally, the research coordinators and program manager will not be blinded to treatment allocation. We do not anticipate the need for unblinding, however, any inadvertent unblinding will be reported.

### Data collection

Data collection will be the responsibility of the research coordinators with oversight from the site principal investigators. Investigators will be responsible for ensuring the accuracy, completeness, legibility, and timeliness of the data collection.

Baseline demographics, medical history, performance status, and disease characteristics will be obtained from EMR review. The remaining data will be gathered from EMR review and surveys administered at baseline (within 1 week of enrollment), 2 months, and 6 months. Research coordinators will administer all surveys by telephone and record responses in the central REDCap database, stored on password protected computers on servers within Johns Hopkins University [37].

### Plans to promote participant retention and complete follow-up

To promote participant retention and complete follow-up, research coordinators will make every effort to remain in contact with study participants throughout their time on the study. If a participant is not reachable within 2 weeks of the baseline, 2-month, and 6-month surveys, the research coordinators will attempt to regain contact through three telephone calls, scheduled visits for clinical care, and if necessary, a letter to the participant’s last known mailing address. These attempts will be documented in the participant’s medical record and study file. If issues with retention occur, these will be brought to the CABs to aid in addressing and correcting these issues.

### Data management

The lead principal investigator and central study team will have access to the data sets from all three enrollment sites. The site principal investigators and research coordinators will only have access to their own site’s data. All electronic data will be stored on password protected computers on servers within Johns Hopkins University. All data collected using paper forms will be stored in a locked filing cabinet within an office assigned to the study team. Study data will be retained in a deidentified manner for 5 years following study completion.

### Confidentiality

We will assign each participant with a study ID, which will be stored securely on REDCap and will only be accessible to the study team. All stored data will be deidentified.

### Plans for collection, laboratory evaluation and storage of biological specimens for genetic or molecular analysis in this trial/future use

This is not applicable to the study.

### Statistical analysis plan

Intention-to-treat analysis will be used with each patient to be included in the group to which they were assigned at randomization regardless of adherence to the intervention or crossover. Descriptive statistics will be performed and presented as means with standard deviations, medians with ranges, or frequencies with proportions, overall and by study arm. To estimate the treatment effect on completion of ACP, we will use a logistic regression model with study arm as the primary predictor and enrollment site and cancer stage (AJCC stage III or IV) as the covariates. The exponentiated coefficient for the study arm will estimate the odds ratio of ACP comparing the intervention arm to the enhanced standard of care arm. Any other covariates that are differentially distributed by study arm will also be included in the model for adjustment. To estimate the treatment effect on QOL, we will use generalized mixed-effects linear regression modeling with a random intercept and robust variance estimate. We will use longitudinal generalized mixed-effects modeling methods for all other secondary outcomes. We will report the effect size, standard error, and 95% confidence interval for the estimate of the treatment effects at 6 months.

### Interim analyses

We do not anticipate significant safety issues associated with the study intervention or participation in the study. However, interim reports of enrollment, outcome completion and safety data will be prepared for the Data and Safety Monitoring Board (DSMB) on a pre-determined schedule. No formal interim analyses for efficacy or futility are planned unless requested by the DSMB.

### Additional analyses

We do not have any additional analyses planned.

### Protocol non-adherence and missing data

We expect missing values to be minimal due to the ability to retrieve the outcome data even for patients who pass away or withdraw from the study through chart review and caregiver-provided information. Missing data on the baseline covariates are expected to be within 5%. Mixed-effects models can account for missing data under the assumption of missing at random (MAR), where it is assumed that missing scores depend on patient covariates in the model as well as the scores at previous time points. In addition, depending on the proportion of losses to follow-up, we will employ a different strategy for sensitivity analyses. If we observe > 5% missing data at the patient-level and an assumption of MAR is plausible, we will perform multiple imputation to address partial data. Missing outcome data will be imputed, and the treatment effect will be estimated under different scenarios to enable comparison of different sensitivity analyses.

### Plans to give access to the full protocol, participant-level data and statistical code

We plan to make the full protocol, deidentified participant-level data, and the statistical code available from the corresponding author on reasonable request.

### Oversight and monitoring

The coordinating center for this trial is Johns Hopkins University, School of Medicine, Department of Surgery, and Division of Surgical Oncology. The trial steering committee meets weekly and is comprised of the lead principal investigator, program manager, research coordinator, and lead CHW, as well as a senior research program coordinator and the clinical research program administrator within the Department of Surgery at Johns Hopkins University.

### Data safety monitoring board

The DSMB is comprised of three independent health services researchers, a community health worker with related expertise, and an unblinded statistician who is not involved in study design or conduct. The DSMB will function independently from the research team throughout the trial to ensure that all study procedures are being performed in accordance with the study protocol. Meetings will be arranged via video conferencing to review protocols, procedures, and concerns related to research integrity. A charter has been established by DSMB members to guide its governance.

### Adverse event reporting and harms

This study does not involve any invasive procedures and as such, there are minimal safety concerns for study participants. Participants will be provided with the lead principal investigator’s contact information and will be advised report any adverse events or potential harms directly to the principal investigator.

### Frequency and plans for auditing trial conduct

Each enrollment site will perform internal quality control of study conduct, data collection, documentation, and completion.

Quality control at the central study site will include the following procedures: (1) the research coordinator will review documentation of the consenting process and completion of the consent documents; (2) the program manager and lead principal investigator will assess data quality on a monthly basis by conducting a random case review of 5% of all clinical data collected that month to assess for missing or incomplete data and excessive variability; (3) the study team will monitor consistent delivery of the study intervention at each enrollment site throughout the trial through monthly meetings and feedback from the lead CHW; (4) the study team will review protocol deviations on an ongoing basis and will implement corrective actions when the quantity or nature of deviations are deemed to be at a level of concern.

### Plans for communicating important protocol amendments to relevant parties

Any significant modifications to the study protocol that could impact study conduct, potential benefits or harms to patients and their caregivers, or participant safety will require a formal protocol amendment. Protocol amendments will be agreed upon by investigators from all enrollment sites and approved by the institutional review board (IRB) at Johns Hopkins University prior to implementation.

### Dissemination plans

The study findings will be presented to the CABs at each enrollment site. We will consider hosting a symposium for all stakeholders, including patients, caregivers, clinicians and other frontline clinical workers, health system leaders, community organizations, advocacy organizations, as well as payors and policymakers. The study findings will also be presented at national and international conferences and a manuscript outlining the study findings will be submitted to a high-impact journal for publication.

## Discussion

The DeCIDE PC intervention utilizes CHWs as palliative care team members with the goal of enhancing palliative care outcomes and reducing disparities for African American patients with advanced cancer. Upon completion of the clinical trial, we will evaluate the implementation of the DeCIDE PC intervention to identify facilitators and barriers to implementation. This will be performed through multilevel analysis of intervention delivery and qualitative interviews with key stakeholders who were involved in implementation of the intervention. We believe this will add insight and context to the summative findings from the effectiveness trial and will contribute to future dissemination efforts.

We expect the DeCIDE PC intervention to improve integration of palliative care, reduce barriers to care, enhance linkage to resources, and improve palliative care outcomes for African American patients with advanced cancer. If found effective, DeCIDE PC may be a transformative model with the potential to guide large-scale adoption of promising strategies to improve palliative care use and will serve as a model for similar action in other academic and community oncology centers.

## Data Availability

The full protocol will be available from the corresponding author on reasonable request. This manuscript does not contain participant-level data.

## Abbreviations

QOL: Quality of life
ASCO: American Society of Clinical Oncology
SDOH: Social determinants of health
CHW: Community health worker
CHWs: Community health workers
DeCIDE PC: Community Health Worker Intervention for Disparities in Palliative Care
ACP: Advance care planning
CAB: Community Advisory Board
CFIR: Consolidated Framework for Implementation Research
RE-AIM: Reach, Effectiveness, Adoption, Implementation, Maintenance
SPIRIT: Standard Protocol Items: Recommendations for Interventional Trials
AJCC: American Joint Committee on Cancer
EMR: Electronic medical record
CAPC: Center to Advance Palliative Care
LW: Living will
DPOA: Durable power of attorney
FACIT-Pal: Functional Assessment of Chronic Illness Therapy - Palliative Care
QOC: Quality of Communication
ESAS: Edmonton Symptom Assessment Scale
CES-D: Center for Epidemiologic Studies Depression Scale
SMD: Standardized mean difference
REDCap: Research Electronic Data Capture
MAR: Missing at random
DSMB: Data and Safety Monitoring Board
IRB: Institutional review board
